# Systemic Injection of Substance P Promotes Murine Calvarial Repair Through Mobilizing Endogenous Mesenchymal Stem Cells

**DOI:** 10.1038/s41598-018-31414-5

**Published:** 2018-08-29

**Authors:** Yueling Zhang, Shu An, Jin Hao, Feng Tian, Xinyi Fang, Jun Wang

**Affiliations:** 1 State Key Laboratory of Oral Diseases, Department of Orthodontics, West China School of Stomatology, West China Hospital of Stomatology, Sichuan University, Chengdu, Sichuan 610041 China; 2 Harvard School of Dental Medicine, Harvard University, Boston, MA 02115 USA; 3 F.M. Kirby Neurobiology Center, Boston Children’s Hospital and Harvard Medical School, Boston, MA 02115 USA

## Abstract

Craniofacial defect is a critical problem in dental clinic, which has a tremendous impact on patients’ quality of life. Mesenchymal stem cell-based therapy has emerged as a promising approach for tissue defect repair. However, reduced survival after mesenchymal stem cells (MSCs) transplantation remains as a major problem in this area, which hampers the outcome of regeneration. Recently, the mechanism to mobilize endogenous MSCs for tissue regeneration has received increasing attentions, as it does not require exogenous cell transplantation. The primary goal of this study was to confirm the role of intravenous substance P in mobilizing endogenous CD45^−^CD11b^−^CD29^+^ MSCs in critical-sized bone defect animals and to investigate the effects of substance P on calvarial bone repair. Flow cytometry analyses revealed that intravenous substance P promoted the mobilization of endogenous CD45^−^CD11b^−^CD29^+^ MSCs after bone defect. In addition, Micro-CT showed that intravenous substance P improved the outcomes of calvarial bone repair. Furthermore, we discovered that systemic injection of substance P attenuated inflammation and enhanced the survival of the local-transplanted GFP^+^ MSCs. Our findings suggested that substance P together with its mobilized CD45^−^CD11b^−^CD29^+^ MSCs helped improve calvarial defect repair through regulating inflammatory conditions and promoting the survival of local-transplanted cells.

## Introduction

The cranium is a highly complex region of human body and deputes to many fundamental functions including eating, speech, expression of emotions, and the delivery of sensations. Therefore, craniofacial defects, as one of the most common problems in oral clinics, can lead to a tremendous impact on patients’ quality of life^1^. The current treatment for craniofacial defects includes autologous tissue grafts, allogeneic tissue grafts, and xenogeneic tissue grafts^2^. However, such procedures bear the risks of donor-site morbidity, poor biocompatibility, and immune rejection^3^. Given the limitations of these tissue graft-based approaches, stem cell-based therapies have emerged as a promising approach for tissue defect repair^4,5^.

Despite recent progress in stem cell transplantation, such method still has several problems, including increased cell death^6,7^ and risk of side effects, such as hypersensitivity to stimuli after transplantation^8^. In addition, a recent study shows that the endogenous pro-inflammatory T cells may impede stem cell transplantation-based bone repair through IFN-γ and TNF-α pathways^9^.

Recently, researchers have found that endogenous MSCs can provide a novel source for defect repair and may overcome the above limitations^1,6–8^. These endogenous MSCs are present in multiple adult tissues and can be recruited to the injury site after stimulation^10,11^. In addition to their multipotency, they have excellent capability of immunomodulation in controlling inflammation after injury, contributing to tissue repair.

The next critical step would be to promote the mobilization of these endogenous MSCs to the injured tissue. Recently, researchers have demonstrated that substance P, as an injury-inducible messenger, can act early in the wound healing process and mobilize CD45^−^CD11b^−^CD29^+^ MSCs from bone marrow into peripheral circulation using corneal injury model^12^.

In this study, we identified whether systemic delivery of substance P can promote endogenous MSCs mobilization and homing in mice with calvarial defects. Next, we evaluated the inflammation state through analyses of pro-inflammatory cytokine expression in both injury sites and peripheral circulation. Finally, we tested the capacity of systemic-injected substance P in promoting calvarial defect repair. Here, we demonstrated that systemic delivery of substance P could promote CD45^−^CD11b^−^CD29^+^ MSCs mobilization and calvarial defect repair. In addition, our study indicated the potential role of systemic-injected substance P in regulating inflammation during bone healing process.

## Results

### Substance P mobilized CD45^−^CD11b^−^CD29^+^ cells

Previous studies had reported that substance P had a strong mobilization effect on the endogenous CD45^−^CD11b^−^CD29^+^ cell population at early stage in corneal burn injury models. These cells all expressed similar molecular markers with BMSCs and had multipotent differentiation capacities at early passages^12^.

**Figure 1:**
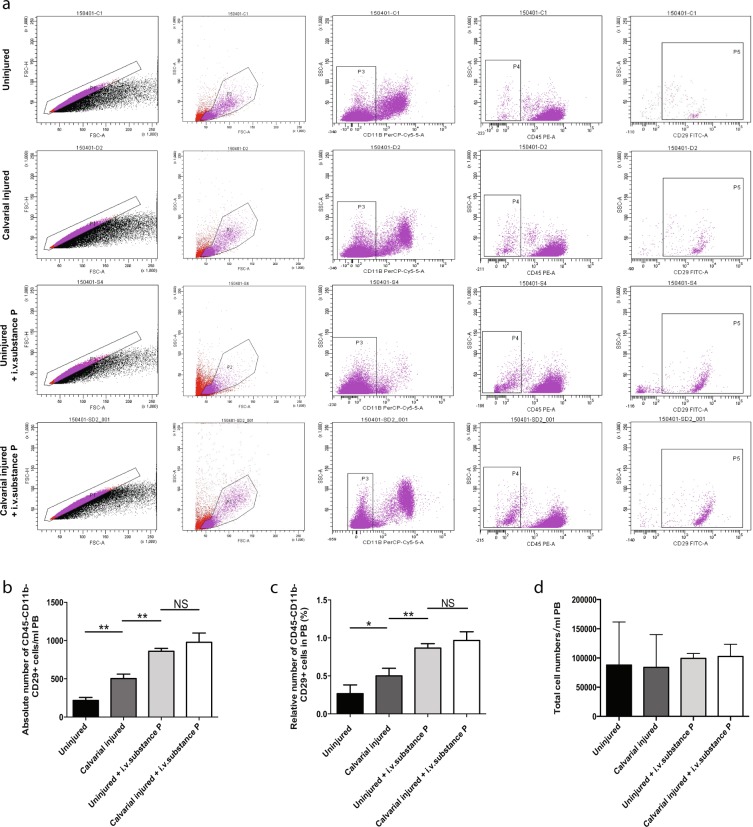
Substance P mobilized CD45^−^CD11b^−^CD29^+^ cells after calvarial defect. C57BL/6 wide-type mice were divided into four groups: uninjured group (no injury), calvarial injured group, uninjured +i.v.substance P, calvarial injured +i.v.substance P. (**a**) 3 days after setting up the animal model, 1 ml peripheral blood was collected from each group for flow cytometry. (**b**) The absolute number of CD45^−^CD11b^−^CD29^+^ cells per 1 ml peripheral blood. (**c**) The relative number of CD45^−^CD11b^−^CD29^+^ cells per 1 ml peripheral blood. (**d**) The total cell numbers per 1 ml peripheral blood. For a, b, c and d, n = 3 for all groups. Analysis of variance *P < 0.05, **P < 0.01. NS indicates not significant.

To identify the function of systemically injected substance P in the bone defect model, we established the calvarial critical-sized defect model with a diameter of 5 mm and gave each mouse a systemic injection of substance P (5 nmol/kg) through the tail vein. 3 days after surgery, we collected 1 ml peripheral blood and counted CD45^−^CD11b^−^CD29^+^ cells using flow cytometry. The absolute numbers of CD45^−^CD11b^−^CD29^+^ cells in peripheral blood from the other three groups (injured or i.v.substance P) were significantly higher than that of the uninjured group (Fig. 1a,b) (P < 0.01). This indicated that both calvarial injury and i.v.substance P could promote the enrichment of CD45^−^CD11b^−^CD29^+^ cells in peripheral blood. Meanwhile, no significant differences were observed between uninjured + i.v.substance P group and calvarial injured +i.v.substance P group (P > 0.05) (Fig. 1b). Both groups exhibited a larger number of cells than the calvarial injured group (P < 0.01) (Fig. 1b), indicating that the motivation capability of i.v.substance P was much greater than injury itself.

To confirm that i.v.substance P helps motivate the CD45^−^CD11b^−^CD29^+^ cell population specifically, we measured the relative number of this cell population together with the total cell number in collected peripheral blood (Fig. 1c,d). The percentage of CD45^−^CD11b^−^CD29^+^ cells in peripheral blood of the calvarial injured group (0.5% ± 0.1) was significantly higher than that of the uninjured group (0.267% ± 0.12) (P < 0.05) (Fig. 1c). There was no significant difference between the uninjured +i.v.substance P group (0.867% ± 0.05) and the calvarial injured +i.v.substance P group (0.967% ± 0.12) (P > 0.05), and both groups had a larger number than that in the calvarial injured and uninjured groups (P < 0.01) (Fig. 1c). In addition, there was no significant difference in the total cell number of peripheral blood among the four groups (P > 0.05) (Fig. 1d).

**Figure 2:**
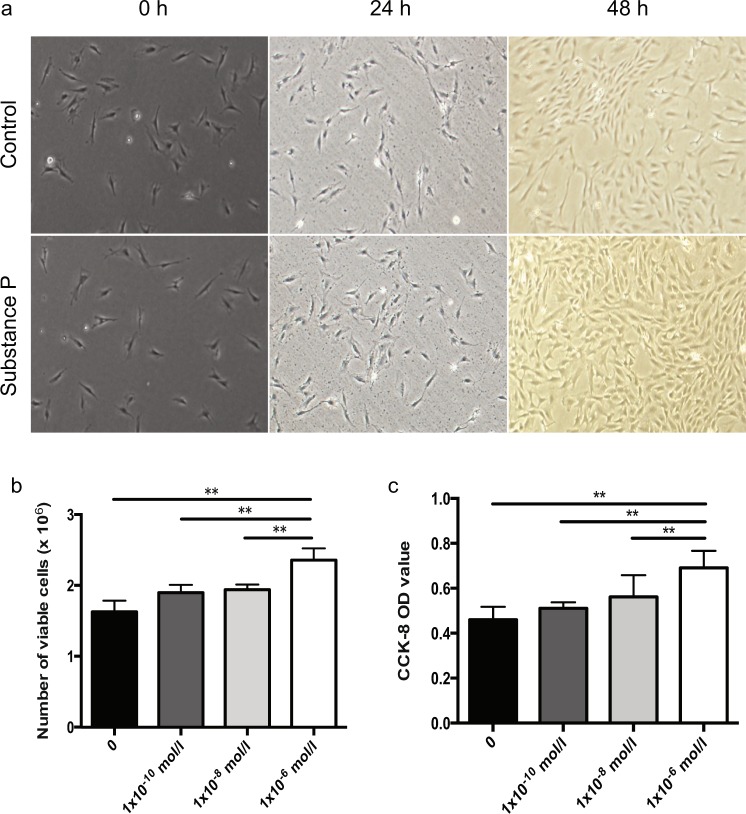
Substance P stimulated the proliferation of CD45^−^CD11b^−^CD29 + cells. (**a**) Representative pictures of cell counting assay for control group and SP group (1 × 10^−6^ mol/l) at 0, 24 and 48 hours respectively. (**b**) Statistical analysis of viable cell numbers within four groups after 48-hour treatment. **(c**) CCK-8 analysis of four groups after 48-hour treatment (0 mol/l, 1 × 10^−10^ mol/l, 1 × 10^−8^ mol/ and 1 × 10^−6^ mol/l). * means P < 0.05. ** means P < 0.01.

We also tested the *in vitro* role of substance P in proliferation of CD45^−^CD11b^−^CD29^+^ cell population using cell counting and CCK-8 (Fig. 2a–c). The statistic analyses have shown that substance P stimulation could increase the proliferation of these mobilized CD45^−^CD11b^−^CD29^+^ cells.

### Intravenous substance P controls inflammatory state both systemically and locally in calvarial injured mice

Substance P plays an important role in neurogenic inflammation and can promote the infiltration of inflammatory cells^13^. Also, substance P can stimulate the secretion of TNF-α from mononuclear-macrophage. To evaluate the inflammatory state of the calvarial injured animal after systemic injection of substance P, we carried out ELISA of the peripheral blood, western blot, and RT-PCR of the tissue within the primary defect areas 2 weeks after the surgery.

**Figure 3:**
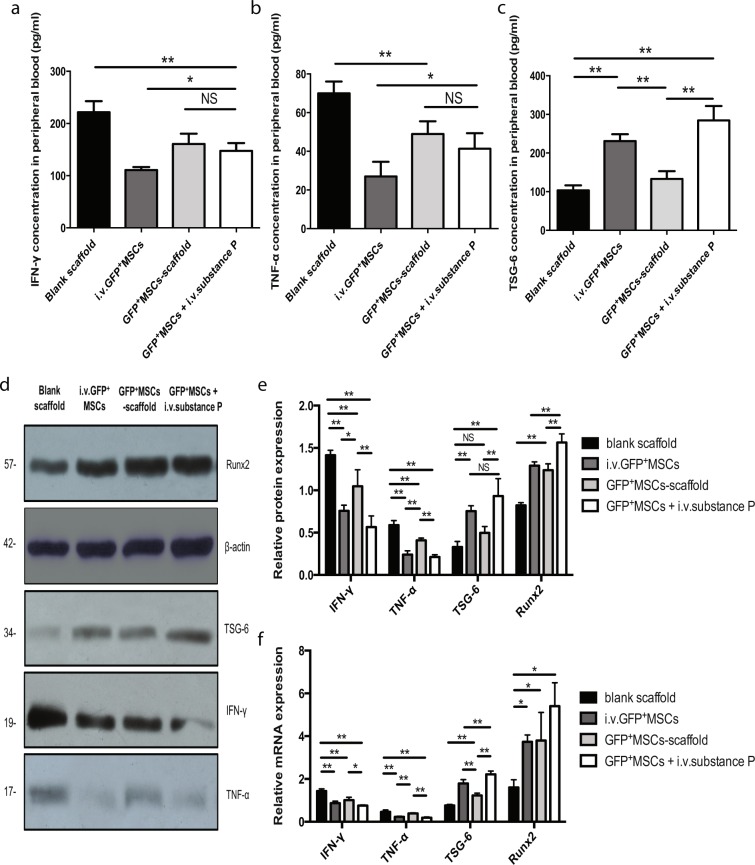
The inflammatory state after intravenous substance P. C57BL/6 wide-type mice were divided into four groups: blank scaffold group, i.v.GFP^+^ MSCs group, GFP^+^ MSCs-scaffold group, GFP^+^ MSCs + i.v.substance P group. 2 weeks post surgery, 1 ml of peripheral blood was harvested from each group for ELISA analysis (n = 6 for each group) and the tissues of the calvarial defect areas were collected for western blot and RT-PCR analysis (n = 3 for each group). (**a**) ELISA analysis for IFN-γ concentration in peripheral circulation. (**b**) ELISA analysis for TNF-α concentration in peripheral circulation. (**c**) ELISA analysis for TSG-6 concentration in peripheral circulation. (**d**) Representative images of western blot for IFN-γ, TNF-α, TSG-6 and Runx2 within defect areas. (**e**) Western blot analysis for relative expression of IFN-γ, TNF-α, TSG-6 and Runx2 at primary injury site. (**f**) RT-PCR analysis for relative mRNA expression of IFN-γ, TNF-α, TSG-6 and Runx2 at primary injury site. Analysis of variance *P < 0.05, **P < 0.01. NS indicates not significant.

First of all, ELISA analyses of peripheral blood have revealed that the inflammation was attenuated with decreased concentration of pro-inflammatory cytokine IFN-γ and TNF-α in peripheral circulation after systemic injection of MSCs or substance P (Fig. 3a,b) (P < 0.05). It has also been reported that TSG-6 can be secreted by intravenous MSCs^14^, which is able to abort the early inflammatory response through the modulation of nuclear factor NF-κB signaling in resident macrophages^15^. In the current study, ELISA analyses have demonstrated that the TSG-6 expression levels in peripheral circulation were significantly higher in groups using intravenous MSCs or substance P than that of the other groups (Fig. 3c) (P < 0.01).

Secondly, western blot analyses of tissue within the primary defect areas have shown that the expression of IFN-γ and TNF-α was significantly reduced after systemic application of MSCs or substance P (Fig. 3d,e) (P < 0.05). RT-PCR analyses of primary injured tissues further confirmed that the corresponding mRNA expressions of IFN-γ and TNF-α were also inhibited in i.v.GFP^+^ MSC and GFP^+^ MSC +i.v.substance P groups (Fig. 3f) (P < 0.05). Additionally, both the mRNA and corresponding protein expression of TSG-6 at the local injury site were enhanced after intravenous MSCs or substance P injection, as shown by RT-PCR and western blot, respectively (Fig. 3e,f) (P < 0.01). Interestingly, the TSG-6 expression level in i.v.GFP^+^ MSC group was similar to that in GFP^+^ MSC +i.v.substance P group (Fig. 3e) (P > 0.05), suggesting that substance P-mobilized CD29^+^ cells, like intravenous exogenous MSCs, could also secrete TSG-6 to help control inflammation.

Recent research has also demonstrated that IFN-γ can inhibit the osteogenesis of exogenous bone marrow MSCs through downregulation of Runx2 pathway and synergistically enhance TNF-α-induced cell apoptosis^9^. Considering that the survival and osteogenic capability of MSCs are essential to bone repair within our calvarial defect models, we wonder whether the Runx2 pathway in defect areas could be modulated by i.v.substance P or i.v.MSCs. To address this critical question, we evaluated the mRNA and protein expression of Runx2 within the primary injury site. The results showed that Runx2 expression increased in GFP^+^ MSC + i.v.substance P group compared with those in other groups (Fig. 3e,f) (P < 0.01). This indicated that the survival and osteogenic capacity of MSCs within the defect in GFP^+^ MSC +i.v.substance P group was higher than those in other groups, which was potentially due to substance P-mediated inflammatory states.

**Figure 4:**
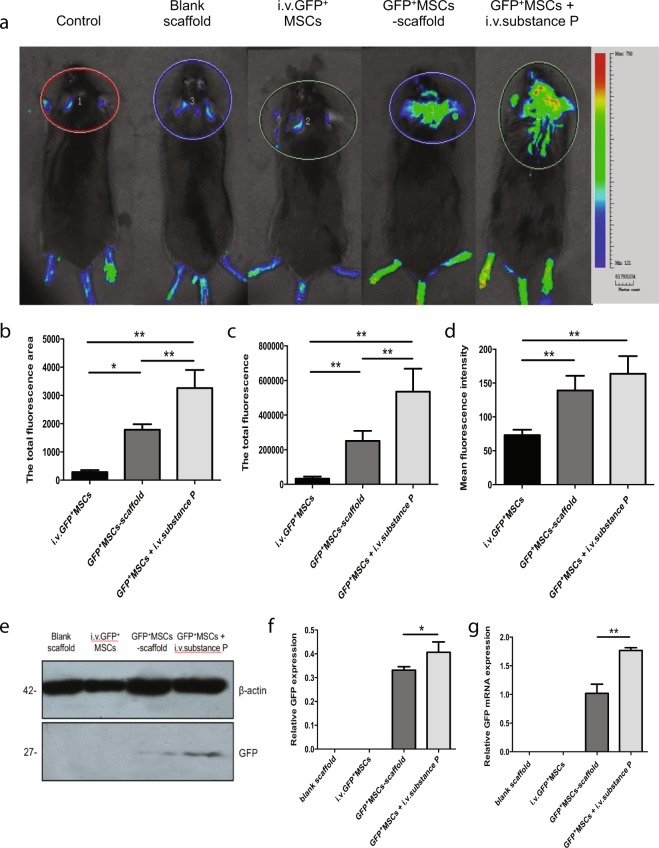
Real-time *in vivo* GFP fluorescence imaging. C57BL/6 wide-type mice were divided into five groups: control group, blank scaffold group, i.v.GFP^+^ MSCs group, GFP^+^ MSCs-scaffold group, GFP^+^ MSCs + i.v.substance P group. (**a**) 12 weeks post surgery, the expression of GFP^+^ signal was detected by Bio-Real *in vivo* imaging system. (**b**) The total fluorescence area for the latter three groups. (**c**) The total fluorescence for the latter three groups. (**d**) Mean fluorescence intensity for the latter three groups. (**e**) Representative images of western blot for GFP within defect areas in the latter four groups. (**f**) Western blot analysis for relative GFP expression within defect areas. (**g**) RT-PCR analysis for relative GFP mRNA expression within defect areas. n = 3 for all groups. Analysis of variance *P < 0.05, **P < 0.01.

To further confirm the effect of substance P-mediated inflammation on survival of transplanted GFP^+^ MSCs, we checked the GFP signal using real-time *in vivo* GFP fluorescence imaging (Fig. 4a–d). The GFP signal was significantly higher in GFP^+^ MSC + i.v.substance P group than in GFP^+^ MSCs-scaffold group (Fig. 4a–d) (P < 0.05), suggesting that i.v.substance P might enhance the survival of GFP^+^ MSCs within the defect areas. We also compared the mRNA and protein expression of GFP in defect areas among groups (Fig. 4e–g). The expression of GFP was only detected within groups using GFP^+^ MSCs-seeded scaffold, confirming that intravenous exogenous GFP^+^ MSCs could hardly reach the injury site^16^. The statistical analyses indicated that the GFP expression was significantly higher in GFP^+^ MSC + i.v.substance P group than in GFP^+^ MSCs-scaffold group (Fig. 4f,g) (P < 0.05), which was consistent with real-time *in vivo* GFP fluorescence imaging data.

All the above results suggested that intravenous substance P could control the inflammatory state both locally and systemically in the calvarial critical-sized defect model.

### Intravenous substance P promoted calvarial injury repair

**Figure 5:**
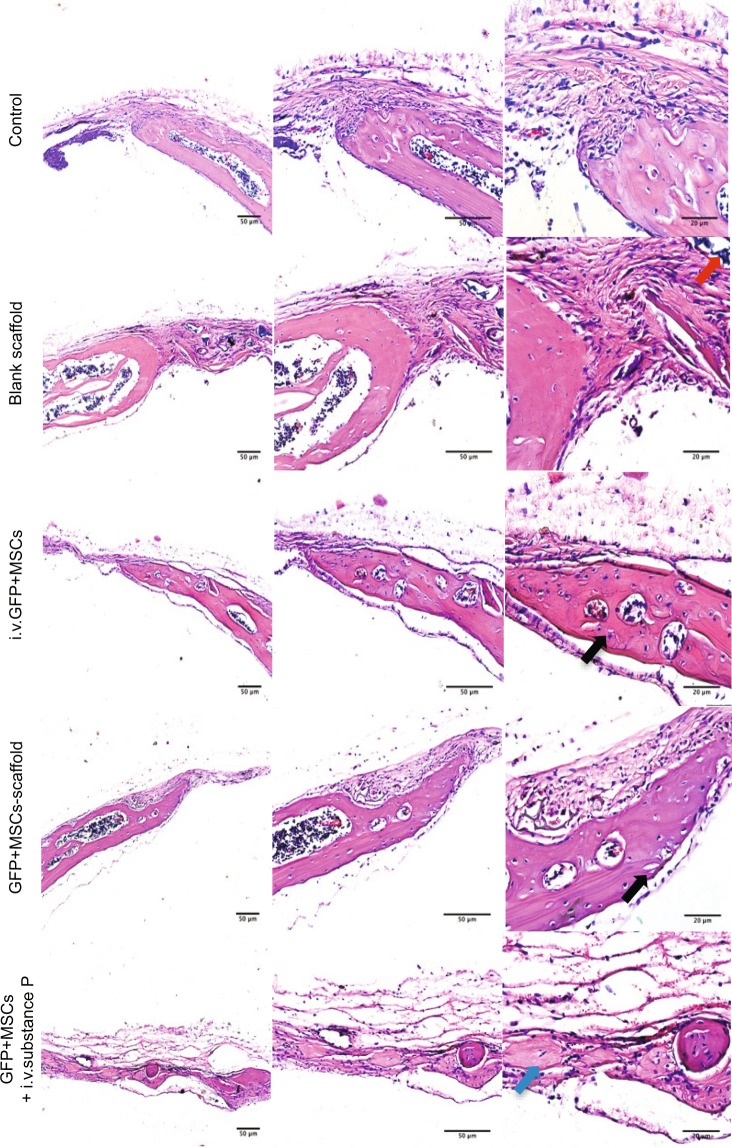
HE staining of calvarial defect areas. C57BL/6 wide-type mice were divided into five groups: control group, blank scaffold group, i.v.GFP^+^ MSCs group, GFP^+^ MSCs-scaffold group, GFP^+^ MSCs + i.v.substance P group. H&E staining of calvarial defect areas for each group. Red arrow indicates the scaffold. Black arrows indicate new bone formation at the margin of calvarial defects. Blue arrow indicates new bone island formation within primary injury site.

**Figure 6:**
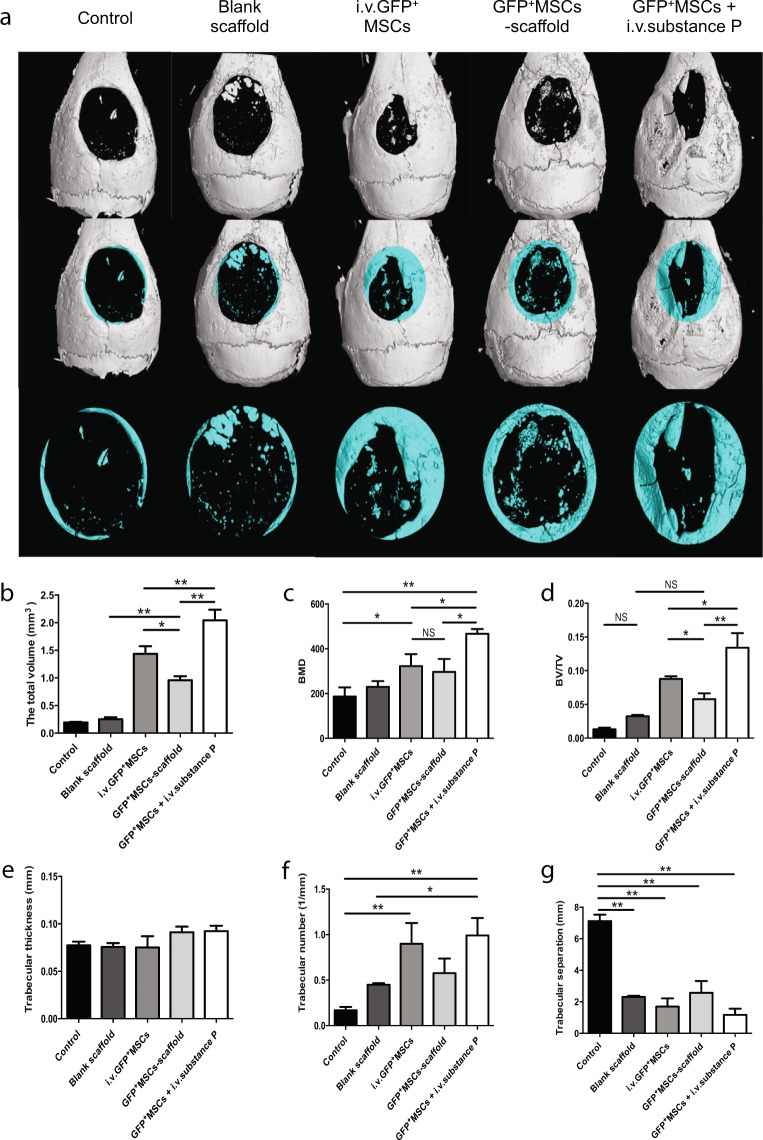
Micro-CT image reconstruction of calvarial defect areas. C57BL/6 wide-type mice were divided into five groups: control group, blank scaffold group, i.v.GFP^+^ MSCs group, GFP^+^ MSCs-scaffold group, GFP^+^ MSCs + i.v.substance P group. (**a**) Micro-CT images and reconstruction of the critical-sized calvarial defects at the 12^th^ week. The blue circular regions on the 2nd line present the configuration of surgery-created defects with a diameter of 5 mm. The blue structures within the circles stand for the new-formed bone-like tissue. The 3rd line is a magnification of the defect area. (**b**) The total volume of new-formed bone. (**c**) The bone mineral density (BMD) of new-formed bone. (**d**) The bone volume fraction (BV/TV): the percentage of the new formed bone in the total calvarial bone volume. (**e**) Mean trabecular bone thickness of new-formed bone. (**f**) The average number of trabecular bones. (**g**) Mean trabecular space within the repair areas. n = 3 for all groups. Analysis of variance *P < 0.05, **P < 0.01. NS indicates not significant.

Considering that substance P can mobilize CD29^+^ cells, which demonstrate multipotent differentiation capacity and can control the inflammatory state after injury, we wonder whether intravenous substance P can promote bone repair in calvarial defects. The hematoxylin-eosin staining showed that there were more bone-like structures formed within the primary defect areas in GFP^+^ MSC + i.v.substance P group compared with other groups (Fig. 5). Consistently, the reconstructed three-dimensional images of Micro CT showed that the remaining defect area after repair was much smaller in GFP^+^ MSC + i.v.substance P group than that of the other groups (Fig. 6a), which suggested that more new bones were formed in GFP^+^ MSC + i.v.substance P group. Further analyses of the bone parameters from Micro CT images suggested that both systemic injection of MSCs and substance P enhanced calvarial bone repair while intravenous substance P brought about better effects than intravenous MSCs in calvarial defect mice (Fig. 6b–g) (P < 0.05).

## Discussion

Recently, researchers have revealed that substance P, as a damage-inducible factor, is a powerful factor for mobilizing endogenous MSCs in corneal burn injury^12^. In our study, we confirmed the ability of substance P in endogenous MSCs mobilization at the early stage of bone injury. These results suggest that substance P may provide a promising candidate for mobilizing endogenous MSCs in different kinds of injury models. Based on previous literature, these substance P-mobilized MSCs may be derived from the bone marrow^12^. Unfortunately, with current techniques, we cannot demonstrate their definite origin. However, we provided some evidence regarding the potential origin of these mobilized cells. On one hand, the molecular markers of substance P mobilized cells are similar to those of BMSCs derived from the bone marrow^12^. On the other hand, the *in vitro* experiment showed that substance P could stimulate the proliferation of those mobilized cells, which was consistent with the effect of substance P on BMSCs^12^. Taking all these together, we speculate that these mobilized cells may be derived from the bone marrow, which may contribute to clavarial bone repairing.

Inflammatory state has been evidenced to be critical for tissue regeneration. Previous studies have focused on the pro-inflammatory roles of substance P as a damage-inducible factor. It has also been reported that substance P can promote the infiltration of inflammatory cells and stimulate secretion of TNF-α from mononuclear-macrophage^13^. In the mouse liver injury model, the inflammation of mice liver was attenuated after administration of NK-1R (a receptor of substance P) antagonists and levels of TNF-α and IFN-γ in serum were also reduced^17^. To identify the effects of intravenous substance P on inflammatory status of our calvarial defect animals, we assessed the pro-inflammatory protein levels both locally and systemically. Interestingly, in the current study, the inflammation was significantly attenuated by systemic infusion of substance P. Previous studies have shown that exogenous substance P has a short half-life while endogenous injured tissue-derived substance P has mostly regressed by Day 3 after injury^12,18^. In this way, the alleviated inflammatory state at 2 weeks after surgery could be due to indirect roles of substance P rather than direct roles. Considering that substance P can mobilize endogenous MSCs, which have immune-regulatory properties^19^, we hypothesized that substance P-mobilized endogenous MSCs may orchestrate the inflammatory properties of substance P itself and help regulate inflammatory responses after calvarial defects. In support of this notion, we also evaluated the TSG-6 expression both systemically and locally. ELISA data showed that anti-inflammatory protein TSG-6 in peripheral blood was induced after intravenous substance P injection. In accordance with this result, TSG-6 expression within primary defect site was also increased. Previous studies have reported that TSG-6 could be derived from MSCs in peripheral blood^14^. Therefore, it is possible that increased TSG-6 in this study could be secreted by substance P-mobilized MSCs, which might contribute to indirect effect of substance P in controlling inflammation.

To boost bone repair after injury, we also combined the systemic injection of substance P with local-transplanted MSCs. We have found that intravenous substance P might improve the survival of local-transplanted MSCs. Considering the immune-regulatory effects of substance P-mobilized endogenous MSCs both locally and systemically, it is possible that systemic infusion of substance P can promote local-transplanted MSCs survival through its mobilized endogenous MSCs. However, more experiment, like *in vitro* cell experiment, is needed to fully elucidate the underlying mechanisms.

In addition, the bone parameters from Micro CT analysis revealed that the new bone formation was enhanced after systemic application of MSCs or substance P. Interestingly, the outcome in GFP^+^ MSC + i.v.substance P group was even better than that in i.v. GFP^+^ MSC group, which might be explained by pro-inflammatory T cell inhibition on exogenous MSCs^9^.

All these results suggest that intravenous substance P can promote bone repair. Similar to our findings, another group has recently promoted bone repair through local application of substance P^20^. However, in our model, we highlighted the combination of systemic infusion of substance P with local-transplanted MSCs in bone repair, which not only took advantage of endogenous MSCs, but also promoted the survival of local-transplanted MSCs. Moreover, we observed that the Runx2 expression within the defect sites, which was associated with osteogenesis of mesenchymal stem cells^9^, was enhanced after intravenous substance P injection, suggesting that systemic infusion of substance P together with its mobilized MSCs could promote osteogenesis of local MSCs in the bone defect animals. All these above results indicate that i.v.substance P can effectively promote bone repair in the calvarial defect mice.

Collectively, our study highlights the effects of substance P in bone repair through mobilizing endogenous MSCs and also indicates the possible roles of intravenous substance P in regulating inflammatory conditions in bone defects. In addition, the combination of intravenous substance P and local-transplanted MSCs treatment can effectively promote the osteogenesis of MSCs and boost calvarial bone repair.

## Methods

### Animal experiments

For the first part of animal experiments, twelve C57BL/6 wide-type mice (10-week-old) were randomly divided into four groups: uninjured, calvarial injured, uninjured + i.v.substance P and calvarial injured + i.v.substance P groups. We established the calvarial critical-sized defect model with a diameter of 5 mm. In uninjured + i.v.substance P and calvarial injured + i.v.substance P groups, each mouse was given a systemic injection of substance P (5 nmol/kg, dissolved in phosphate-buffered saline) through the tail vein. 3 days after surgery, we collected 1 ml peripheral blood and counted CD45^−^CD11b^−^CD29^+^ cells using flow cytometry.

For the second part of animal experiments, thirty C57BL/6 wide-type mice (10-week-old) were randomly divided into five groups: control, blank scaffold, i.v.GFP^+^ MSCs, GFP^+^ MSCs-scaffold and GFP^+^ MSCs + i.v.substance P groups. We established the calvarial critical-sized defect model with a diameter of 5 mm. In blank scaffold group, we put the 5 × 5 mm^2^ gelatin sponge within calvarial defect area. In i.v.GFP^+^ MSCs group, we gave each mouse a systemic injection of 5 × 10^6^ GFP^+^ MSCs (dissolved in 200 μl phosphate-buffered saline) through its tail vein. In GFP^+^ MSCs-scaffold group, the 5 × 5 mm^2^ gelatin sponge seeded with GFP^+^ MSCs was set in calvarial defect area of each mouse. In GFP^+^ MSCs + i.v.substance P groups, we put one GFP^+^ MSCs-seeded scaffold in calvarial defect area and gave each mouse a systemic injection of substance P (5 nmol/kg, dissolved in phosphate-buffered saline) through the tail vein.

### Flow cytometry

3 days after surgery, 1 ml peripheral blood was harvested from calvarial injured mice (uninjured, calvarial injured, uninjured + i.v.substance P and calvarial injured + i.v.substance P groups). Each sample from an individual mouse was separately prepared and incubated with the antibodies CD29 (BioLegend, San Diego, CA), CD11b (BioLegend, San Diego, CA), CD45 (BioLegend, San Diego, CA) for 30 minutes at 4 °C before flow cytometry analysis. FlowJo was used for flow cytometric analyses.

### Cell counting and CCK8

Substance P-mobilized CD29^+^ cells were isolated by MACS sorting (Miltenyi) from the peripheral blood at 3 days after surgery (uninjured, calvarial injured, uninjured + i.v.substance P and calvarial injured + i.v.substance P groups). All the single cells were seeded at 1 × 10^6^ into 100 mm culture dishes (Corning, NY, USA) and incubated in α-MEM medium supplemented with 10% fetal bovine serum, 100 U/mL penicillin, and 100 μg/mL streptomycin (all from Gibco, Grand Island, NY, USA) at 37 °C and 5% CO_2_. Medium was changed after 3 days. Then we treated these cells with different doses of substance P (0 mol/l, 1 × 10^−10^ mol/l, 1 × 10^−8^ mol/ and 1 × 10^−6^ mol/l). After 48 hours of treatment, cell counting was performed.

CCK8 was also carried out. We seeded 4 × 10^3^ CD29^+^ cells in each well of 96-well plate in 100 μl of culture medium and pre-incubated the plate for 24 hours at 37 °C and 5% CO_2_. Then the cells were treated with 1 × 10^−10^ mol/l, 1 × 10^−8^ mol/ and 1 × 10^−6^ mol/l of substance P in three experiment groups respectively. After 48 hours of treatment, 10 μl of CCK8 solution (Dojindo, Tabaru, Japan) was added to each well of the plate. After 2-hour incubation at 37 °C and 5% CO2, the optical density (OD) value of each well was measured at 450 nm using a microplate reader (Bio-Rad, Hercules, CA, USA).

### Cell isolation and culture

MSCs from C57BL/6 wide-type and green fluorescent protein positive (GFP^+^) mice were isolated and cultured *in vitro* respectively. Bone marrow cells were flushed out from bone cavity of femurs and tibias of 6-week old C57BL/6 wide-type and GFP^+^ mice with PBS. A single-cell suspension of all nucleated cells was obtained by passing all bone marrow cells through a 70-μm cell strainer (BD Bioscience). All the single cells were seeded at 1 × 10^6^ into 100-mm culture dishes (Corning) and initially incubated for 48 h at 37 °C and 5% CO2. The attached cells were cultured with alpha minimum essential medium (α-MEM, Invitrogen) supplemented with 20% FBS, 2 mM L-glutamine (Invitrogen), 55 μM 2-mercaptoethanol (Invitrogen), 100 U ml^-1^ penicillin, and 100 g ml^-1^ streptomycin (Invitrogen). To eliminate the non-adherent cells, the cultures were washed with PBS twice on the second day. Then, the medium was changed every 2~3 days. When the confluence reached 70~80%, the cells were passaged to P1.

### Scaffold processing

Under sterile conditions, the gelatin sponge was cut into a size of about 5 × 5 mm^2^ and incubated in 48-well plates with culture medium for 12 hours; after that, the medium was discarded; then P1 MSCs were seeded in the 48-well plates with a number of 1 × 10^6^ within 100 μl medium per well. After incubation for 4–6 hours, 500 μl medium was added to each well. These prepared scaffolds were used in the second part of animal experiments.

### ELISA

2 weeks after the surgery, 1 ml peripheral blood was harvested from each mouse in five groups (control, blank scaffold, i.v.GFP^+^ MSCs, GFP^+^ MSCs-scaffold and GFP^+^ MSCs + i.v.substance P groups) and set stable for 30 min. After centrifugation at 3000 r.p.m. at 4 °C for 10 minutes, the supernatant was collected and assayed by ELISA for IFN-γ (Abcam, Cambridge, UK), TNF-α (Abcam, Cambridge, UK) and TSG-6 (Santa Cruz Biotechnology, Dallas, TX).

### Western blot

2 weeks post surgery half of the mice from each group (control, blank scaffold, i.v.GFP^+^ MSCs, GFP^+^ MSCs-scaffold and GFP^+^ MSCs + i.v.substance P groups) were sacrificed. Half of the tissue within the calvarial injury site from each sacrificed mouse was collected and washed with PBS, and then grinded in liquid nitrogen. Lysed in RIPA buffer (KeyGEN Biotech, China) and then put on ice for 30 min. Centrifuged for 20 min at 4 °C, 12000 g. Then, the supernatant was collected for the whole protein extraction. The membranes were blotted with primary antibodies for IFN-γ (Abcam, Cambridge, UK), TNF-α (Abcam, Cambridge, UK), TSG-6 (Santa Cruz Biotechnology, Dallas, TX), GFP (Cell Signaling, Danvers, MA) and Runx2 (Abcam, Cambridge, UK). After primary antibody incubation, the membranes were washed and incubated for 1 h in the peroxidase-conjugated anti-mouse or rabbit secondary antibody (ZSGB-BIO, China, 1:5000). The relative expression of the tested protein was quantitatively analyzed by the ratio of the gray value between the target protein and β-actin in the same sample.

### Quantitative real-time PCR

For RNA extraction, the other half of the tissue within the calvarial injury site from each sacrificed mouse was collected, minced into small pieces, lysed in RNA isolation reagent (Trizol; Gibco, US), and homogenized using a motor-driven homogenizer. The total RNA was extracted using RNAiso Plus (TaKaRa, Japan). First-strand cDNA was synthesized from 1 μg total RNA using a Reverse Transcriptase PCR Kit (Thermo, Germany). Real-time amplification was performed using Applied Biosystems Prism 7900HT Sequence Detection System (Thermo, Germany). The primers are listed in Table 1.

**Table 1:** Primer sequence for RT-PCR.

Gene	Primer sequence (5′– > 3′)
IFN-γ	(F) GAGTATTGCCAAGTTTGAGGT
	(R) CAGCGACTCCTTTTCCGCT
TNF-α	(F) CACGTCGTAGCAAACCACCAA
	(R) GTTGGTTGTCTTTGAGATCCAT
TSG-6	(F) TGACCTTGAACATGATCCAG
	(R) CTTCAAGGTCATGACATTCCT
Runx2	(F) CAAGAGTTTCACCTTGACCAT
	(R) GTCATCAAGCTTCTGTCTGTG
GFP	(F) CCACATGAAGCAGCACGACT
	(R) GATGCGGTTCACCAGGGTGT

### Real-time *in vivo* GFP fluorescence imaging

12 weeks post surgery, the expression of GFP^+^ signal from each group (control, blank scaffold, i.v.GFP^+^ MSCs, GFP^+^ MSCs-scaffold and GFP^+^ MSCs + i.v.substance P groups) was detected by Bio-Real *in vivo* imaging system (Bio-Real, QuickView3000, Austria). The wavelength of excitation light was 474 nm, and the wavelength of emitted light was 525 nm.

### Micro-CT scanning with image reconstruction

12 weeks after surgery, the remaining mice from each group (control, blank scaffold, i.v.GFP^+^ MSCs, GFP^+^ MSCs-scaffold and GFP^+^ MSCs + i.v.substance P groups) were sacrificed and the calvarial bones were dissected and fixed in 4% paraformaldehyde. Micro-CT scan and image reconstruction were performed by VGstudio Max2.1 software. The total volume, relative bone volume (BV/TV), bone mineral density (BMD), trabecular number (Tb.N), trabecular thickness (Tb.Th), trabecular separation (Tb.Sp) were analyzed.

### Hematoxylin-eosin staining

After fixation in 4% paraformaldehyde and Micro-CT scan, the calvarial bones were then decalcified with 5% ethylenediaminetetracetic acid (EDTA, pH 7.4), followed by paraffin embedding. Coronal sections of 5 μm thickness were obtained. Hematoxylin-eosin stainings were performed for microscopic observation of the sections.

### Statistics

SPSS 20.0 was used to do the statistical analyses. Significance was assessed using an independent two-tailed Student’s t test or with analysis of variance. P < 0.05 was considered significant.

### Ethical Approval

Ethical approval to report this case series was obtained from Ethics Committee of West China School of Stomatology (approval number: WCCSIRB-D-2014-020).

### Statement of human and animal rights

All procedures in this study were conducted in accordance with the Ethics Committee of West China School of Stomatology’s approved protocols (WCCSIRB-D-2014-020). This article does not contain any studies with human subjects.

## Data Availability

All data generated or analyzed during this study are included in this published article.
